# Leptin and leptin receptor polymorphisms are associated with poor outcome (death) in patients with non-appendicular secondary peritonitis

**DOI:** 10.1186/cc10467

**Published:** 2011-09-23

**Authors:** Rodolfo L Bracho-Riquelme, Verónica Loera-Castañeda, Alejandro Torres-Valenzuela, Guadalupe A Loera-Castañeda, J Pablo Sánchez-Ramírez

**Affiliations:** 1 Instituto de Investigación Científica, Universidad Juárez del Estado de Durango, Calle Constitución 404 sur, Durango, Dgo. C.P. 34, 000, México; 2 Servicio de Cirugía, Hospital General de Durango (SSD), Calle 5 de Febrero esq. con C. Norman Fuentes s/n, Durango, Dgo. C.P. 34, 000, México; 3 Laboratorio de genómica aplicada, CIIDIR-IPN Unidad Durango, Calle Sigma 119, Durango, Dgo. C.P. 34, 220, México; 4 Facultad de Medicina y Nutrición, Universidad Juárez del Estado de Durango, Calle Constitución 404 sur, Durango, Dgo. C.P. 34, 000, México

## Abstract

**Introduction:**

Leptin (LEP) and its receptor (LEPR) participate in the immunological response during infection. LEP serum levels rise during sepsis. In patients with peritonitis, an insufficient elevation in serum LEP is associated with an increased risk of death. As gene variants of *LEP *and *LEPR *have been associated with diverse pathologic conditions, we explored the association of genetic polymorphisms of *LEP *or *LEPR *with death in patients with secondary peritonitis.

**Methods:**

A case control study was undertaken. *LEP *Gene -2548G > A and the *LEPR *Gene 223A > G polymorphism were determined in 74 patients. The odds ratio of genotype and allele distribution in survival (control) versus death (case) among patients was calculated. Serum LEP, interleukin (IL)-6, tumour necrosis factor alpha, C-reactive protein (C-RP), IL-10 and IL-13 levels were analyzed in 34 patients.

**Results:**

There were significant differences in genotype and allele distribution between survivors and non-survivors for -2548G > A and 223A > G polymorphisms. The presence of the mutant allele A, in -2548, had an odds ratio of 4.64 (95% CI 1.22, 17.67) with significance (*P *= 0.017) in the risk of death. The presence of mutant allele G, in 223, had an odds ratio of 3.57 (95% CI 1.06, 12.01) with significance in the risk of death (*P = *0.033). The presence of allele A in the -2548 polymorphism was associated with differences in serum LEP (*P = *0.013), and IL-10 (*P = *0.0001). The presence of allele G in 223 polymorphism was likewise correlated with differences in serum LEP (*P *< 0001), C-RP (*P = *0.033), and IL-10 (*P = *0.043).

**Conclusions:**

The polymorphisms studied are associated with death in patients with peritonitis of non-appendicular origin. This association is stronger than many known risk-factors related to peritonitis severity, and is independent of body mass. The physiopathologic mechanism is possibly related to an insufficient increase in the elevation of serum *LEP *levels, and is unrelated to body mass.

## Introduction

Leptin (LEP) is a cytokine-like hormone that is primarily synthesized and secreted by white adipose tissue. As a hormone it has important functions in the regulation of body weight, reproduction, immune functions, bone formation and growth [1]. The biological activities of LEP on target tissues are carried out through selective binding to a specific receptor. Leptin receptors (LEPR) form homodimers that, in general, act by signaling via the Janus kinases to regulate multiple events in target tissues [2].

LEP and LEPR participate in the immunological response during infection. LEP displays both pro- and anti-inflammatory properties. It appears necessary for the induction of interleukin (IL)-6 and tumour necrosis factor alpha (TNF-α) after a lipopolysaccharide (LPS) challenge, while at the same time it provides protection against LPS- or TNFα-induced lethality [3,4]. In humans, LEP levels are higher during sepsis [5-7]. In surgically treated patients with peritonitis, an insufficient rise in serum LEP (< 10 ng/mL) is associated with a threefold-increase in the risk of death (sensitivity 94.4, specificity 50%) [8].

Gene variants of *LEP *and *LEPR *have been associated with obesity, non-Hodgkin's lymphoma, non-insulin-dependent diabetes mellitus, and essential hypertension [1,9-12]. The purpose of this study was to explore the association of genetic polymorphisms of *LEP *or *LEPR *with an unfavourable outcome (death) in patients with non-appendicular secondary peritonitis.

In an attempt to explain the low specificity of the above mentioned LEP threshold, we hypothesized that *LEP *and *LEPR *polymorphisms cause problems with ligand-receptor binding, contributing to an unfavourable outcome.

A positive association between *LEP *or *LEPR *polymorphisms and outcome; serum TNF-α, IL-6 (pro-inflammatory cytokines), IL-10 and IL-13 (anti-inflammatory cytokines), C-RP (acute phase protein), and LEP, illustrating the overall acute phase response, would give us an insight into the causes of the unfavourable outcome.

## Materials and methods

### Patients

Eligible patients were ≥18 years old, with surgically confirmed secondary peritonitis, defined as a polymicrobial infection of the peritoneum occurring as a consequence of traumatic, infectious, ulcerative, obstructive or neoplastic pathology [13]. All were admitted to the Department of General Surgery of the Hospital General de Durango (Mexico). Excluded were patients with any condition causing immune-depression, current treatment with an immune-suppressive drug, and peritonitis of appendicular origin. Appendicular peritonitis was excluded because it generally has a favourable outcome [14,15]. The case-group (non-survivors) included patients that died after 24 h and within the 30-day postoperative period. The control-group (survivors) was comprised of those who survived the 30-day follow-up. The Ethics Committee at the Hospital General de Durango (SSD) approved the study. Signed informed consent was obtained from each patient.

The 74 patients recruited consisted of all consecutively eligible patients. The sample was prospective. The first of the patients also participated in a published study, and the last 40 participated exclusively in this protocol [8].

The severity of peritonitis was determined using the Mannheim Peritonitis Index (MPI). The score takes in to account eight adverse factors: age > 50 years (5 points), female gender (5 points), multiple organ failure (MOF) (7 points), neoplasia (4 points), > 24 h of evolution (4 points), non-colonic origin (4 points), generalized peritonitis (6 points), with purulent (6 points) or fecaloid (12 points) character. The MPI classifies severity in intervals: grade I (< 21 points), grade II (21 to 29 points), and grade III (> 29 points). It is a simple scoring system, widely used, and is specific to peritonitis. With this index, the grade-response relation between severity of peritonitis and the concentration of key regulatory serum cytokines has been demonstrated [16].

To assess the patients' nutritional status, the Body Mass Index (BMI) was used. Each individual was classified as follows: underweight (< 18.5 kg/m^2^), normal (18.5 to 24.9 kg/m^2^), grade I overweight (25.0 to 29.9 kg/m^2^), obese or grade 2 overweight (30.0 to 39.9 kg/m^2^), grade 3 overweight or morbid obese (> 40 kg/m^2^) [17].

In accordance with accepted practice principals, the attending physician determined the treatment in each case: correcting fluid and electrolyte deficiencies with parenteral fluids; physiologic organ system support; surgical treatment (source control, peritoneal lavage and drainage) during open exploratory laparotomy along with broad-spectrum antibiotic therapy [18,19].

### Procedures

#### DNA extraction and LEP, LEPR genotyping

DNA was extracted from leukocytes using the DTAB/CTAB method previously described [20]. Gene amplification was performed by the conventional polymorphism chain reaction (PCR) using the following primers: for the *LEP *gene: forward 5'TTTCCTGTAATTTTCCCGTGAG-3' and reverse 5'AAAGCAAAGACAGGCATAAAAA-3'. Amplification of a 242 bp fragment ensued under the following reaction conditions: initial denaturing at 95°C for five minutes and 30 amplification cycles of 94°C, 58.5°C and 72°C for one minute and a final extension at 72°C for five minutes. For the *LEPR *gene, we used the following primers: forward 5'AAACTCAACGACACTCTCCT-3' and reverse 5'TGAACTGACATTAGAGGTGAC-3'. This gave rise to a product of 80 bp using the following amplification conditions: 94°C × 2 minutes of initial denaturing, followed by 32 cycles of 94°C for 1 minute, 50°C for 45 sec and 72°C for 30 sec, with a final extension of 7 minutes. After PCR all products were stored at 4°C.

For the restriction fragment length polymorphism (RFLPs), these PCR products were digested with restriction enzymes, using *Cof*I Promega^® ^(Madison, WI. USA) 1.0 U per 15 μL of the product of PCR was incubated at 37°C for 60 minutes for the -2548G > A polymorphism in the *LEP *gene, generating a restriction pattern of 61 bp and 181 bp when the cleavage site is present. On the other hand the enzyme *Msp*I Sigma^® ^(Saint Louis, MO. USA) 1.5 U per 15 μL of the product of PCR was incubated at 37°C for 60 minutes was used for polymorphism 223A > G of the *LEPR *gene, which generates a cleavage site that produces 22 bp and 58 bp fragments. RFLPs were visualized by means of 2% agarose gel electrophoresis stained with ethidium bromide [20].

A total of S 1.5 U per 15 μL of the product of PCR, incubated at 37°C for 60 minutes, was used for polymorphism 223A > G of the *LEPR *gene, which generates a cleavage site that produces 22 bp and 58 bp fragments. RFLPs were visualized by means of 2% agarose gel electrophoresis stained with ethidium bromide [20].

#### Cytokine and C-Reactive protein analysis

Serum LEP, IL-6, TNF-α, C-RP, IL-10 and IL-13 levels were available for the first 34 patients. The blood samples were taken 18 to 24 h into the postoperative period, irrespective of the time of day. It is during this interval that LEP increases to its maximum postoperative value [21-23].

The samples were allowed to clot, and serum was separated by centrifugation and cryopreserved at -40°C, until the time of cytokine and C-RP determination. Thirty-four samples were processed to determine: LEP (ng/ml), TNF-α (pg/ml), interleukins 6, 10 and 13 (pg/ml), and C-RP (μg/100 ml). Each analyte was evaluated separately using standard ELISA methods: IL-6 and IL-10 were determined with Quantikine^® ^HS (R&D Systems, Minneapolis, MN, USA), LEP, IL-13, and TNF-α with Quantikine^® ^kits (R&D Systems). C-RP was determined with the AlerChek kit for C-RP (AlerCHEK™, Portland, ME, USA).

### Statistical analysis

For the sample size calculation we took into consideration frequencies of 77.4% and 70.14% for polymorphisms -2548G > A, and 223A > G respectively, in accordance with Wang *et al. *in patients with morbid obesity and resistance to insulin [24]. Therefore, an average prevalence of 75% was considered adequate to calculate our sample size, with a variation up to a 10%. Using program EPIDAT (formula *n *= Z^2 ^pq/d^2^) we calculated a sample *n *= 73.

Categorical variables were reported as counts, percentages and 95% CI, and continuous variables as means ± SD, median and percentiles. For categorical variables, the chi-square test was used to assess differences. For continuous variables, a t-test and ANOVA were employed. Odds ratios (OR) and confidence intervals were calculated. *P*-values less than 0.05 indicated statistical significance. Data were collected and analyzed using the SPSS V. 15.0.1 (SPSS Inc. Chicago, IL, USA, 2006) and Epidat 3.1 (Xunta de Galicia/PHO, A Coruña, Galicia, España, 2006) software.

## Results

### Study population

The case-control population comprised 74 adults (41 males and 33 females, mean age: 54.7 ± 21.1 years). The non-survivors were 22 patients (10 males and 12 females, mean age: 68.5 ± 14.6 years). The survivors were 52 patients (31 males and 21 females, mean age: 48.8 ± 20.8 years). The mean hospital stay for all patients was 7.15 ± 5.73 days. A mean BMI of 24.75 ± 3.82, along with the low total proteins, albumin, and A/G ratio (Table 1), suggest malnutrition. A low mean erythrocyte count, haemoglobin, and hematocrit coincide with this appraisal. The mean MPI of the case-control population was 23.12 ± 9.10 points. A high white blood cell count, with bandemia, along with high total bilirubin and direct bilirubin (Table 1), reflect the severity of the sepsis among the study subjects.

**Table 1:** Mean laboratory findings in 74 patients with non appendicular secondary peritonitis

Study	Mean	Reference value	95% confidence interval
Erythrocytes	**4.22 ± 1.11 × 10**^**6**^	4.5 to 6.0 male, 4.2 to 5.4 × 10^6 ^female	(3.76, 4.68)
Hemoglobin	12.79 ± 3.47 g/dL	12 to 17 male, 11 to 15 g/dL female	(11.36, 14.22)
Hematocrit	38.55 ± 10.31%	41 to 52 male, 38 to 46% female	(34.29, 42, 81)
Neutrophils	61.76 ± 27.27%	50 to 70%	(50.50, 73.02)
Bands	**15.92 ± 15.94%**	0 to 3%	(9.34, 22.50)
Platelets	288, 543 ± 160, 829 plts.	150 to 350 × 10^3^	(222, 156.05, 354, 929.95)
Total proteins	**5.49 ± 1.11 g/dL**	6.5 to 8.0 g/dL	(5.23, 5.76)
Albumin	**2.46 ± 0.77 g/dL**	3.5 to 5.0 g/dL	(2.28, 2.65)
Globulins	3.03 ± 0.63 g/dL	2.5 to 3.5 g/dL	(2.88, 3.18)
A/G ratio	**0.82 ± 0.27**	1.3 to 2.5	(0.76, 0.89)
Total bilirubin	**1.42 ± 1.8 g/dL**	up to 1.1 g/dL	(1.00, 1.84)
Direct bilirubin	**0.788 ± 1.66 g/dL**	up to 0.25 g/dL	(0.40, 1.18)
Indirect bilirubin	0.645 ± 0.49 g/dL	0.1 to 0.5 g/dL	(0.53, 0.76)

The sources of secondary peritonitis were the small intestine 24.32% (*n *= 18), gallbladder and biliary tree 17.56% (*n *= 13), colon 16.21% (*n *= 12), stomach 16.21% (*n *= 12), liver 13.51% (*n *= 10), uterus and fallopian tubes 4.05% (*n *= 3), duodenum 4.05% (*n *= 3), pancreas 1.35% (*n *= 1), spleen 1.35% (*n *= 1), and retroperitoneum 1.35% (*n *= 1).

### Clinical characteristics

Comparing survivors and non-survivors, there was a difference in age (*P *= 0.0001), but there was neither a difference in sex-ratio (*P *= 0.3874), nor in hospital stay in days (*P *= 0.828). With respect to BMI categories of underweight (*n *= 0 *vs*. 3), normal (26 *vs*. 11), grade I overweight (20 *vs*. 6), and obese (6 *vs*. 2), there was no difference (*P *= 0.052) between controls and cases. There were differences in total proteins (*P *= 0.006), albumin (*P *= 0.0001), and in the A/G ratio (*P *= 0.0001), between survivors and non-survivors, and in MPI scores (*P *< 0.0001).

Comparing the MPI adverse factors between each group (Table 2), a difference was found for age > 50 years (*P *= 0.0076), generalized peritonitis (*P *= 0.0483), the presence of MOF (*P *< 0001), and coexisting neoplasia (*P *= 0.0007).

**Table 2:** Mannheim Peritonitis Index adverse factors present in the 74 patients with non-appendicular secondary peritonitis

Characteristic	Survivors (n (%))	Non-survivors (n (%))	Total (n (%))	*P*
Age > 50 years	26 (50)	19 (86.4)	45 (60.8)	**0.0076**
Female gender	21 (40.4)	12 (54.5)	33 (44.6)	0.3874
Generalized peritonitis	31 (60)	19 (86.4)	50 (67.6)	**0.0483**
Duration > 24 h	44 (85)	21 (95.5)	65 (87.8)	0.3603
Purulent/fecal character	26 (50)	17 (77.3)	43 (58, 1)	0.0554
Non-colonic origin	44 (85)	15 (68.2)	59 (79.7)	0.1967
Presence of organ failure	4 (7.7)	14 (63.6)	18 (24.3)	**< 0.0001**
Malignant disease	1 (1.9)	7 (31.2)	8 (10.8)	**0.0007**
MPI I	2 (9.1)	38 (73.1)	40 (54.1)	0.0000
MPI II	8 (36.4)	8 (15.4)	16 (21.6)	0.0901
MPI III	12 (54.5)	6 (11.5)	18 (24.3)	0.0003

### Comparison of genetic characteristics

The genotypic distribution of the alleles represented by the polymorphism of the leptin gene at -2548 was in Hardy-Weinberg equilibrium (*P*-value of two-tailed chi-square 0.113807), while the genotype distribution of polymorphism 223 was in disequilibrium (*P*-value of two tailed chi-square 0.023312).

#### -2548 polymorphism

Analysis of the allele distribution in survival versus death among patients with non-appendicular secondary peritonitis (Table 3) showed a higher frequency of the A allele among those with an unfavourable outcome (*P *= 0.002). Considering a dominant model, an odds ratio of 4.64 (95% CI 1.22, 17.67) with statistical significance (*P *= 0.017) was found using contingency tables with A/A + G/A in the rows as the presence of an adverse factor versus G/G, and in the columns, survival versus death.

**Table 3:** The genotype and allele distribution among patients with non appendicular secondary peritonitis

-2548 polymorphism	Survivors (n (%))	Non-survivors (n (%))	*P*
Genotypes			**0.014**
GG	22 (42.3)	3 (13.6)	
GA	21 (40.4)	9 (40.9)	
AA	9 (17.3)	10 (45.5)	
Alleles			**0.002**
G	65 (62.5)	15 (34.1)	
A	39 (37.5)	29 (65.9)	
			
223 polymorphism			
Genotypes			0.072
GG	11 (21.15)	9 (40.9)	
GA	18 (34, 62)	9 (40.9)	
AA	23 (44.23)	4 (18.2)	
Alleles			**0.011**
G	40 (38.5)	27 (61.4)	
A	64 (61.5)	17 (38.6)	

#### 223 polymorphism

Analysis of the allele distribution (Table 3) in the controls versus cases showed a non-significant trend toward higher frequency of the G allele among non-survivors (*P *= 0.011). Analyzing G/G + G/A, as an adverse factor versus A/A, while comparing the outcome, survival versus death, showed a difference, (*P *= 0.033) with an odds-ratio of 3.57 (95% CI 1.06, 12.01).

### Association of genetic polymorphisms with clinical characteristics

Other than death, with an odds-ratio of 4.6 (*P *= 0.017) for -2548 polymorphism, and with an odds-ratio of 3.6 (*P *= 0.0334) for 223 polymorphism, no other clinical variable was found associated with either genetic polymorphism (Table 4).

**Table 4:** Relationship between polymorphisms and clinical variables studied in patients with non-appendicular secondary peritonitis.

		-2548				223			
Variable	Adverse factor/Non-adverse	Carriers	Non-carriers	*P*	OR (IC95)	Carriers	Non-carriers	*P*	OR (IC95)
Age	> 50 years	33	12	0.1069	2.2 (0.83, 5.99)	32	13	0.0908	2.3 (0.87, 6.08)
	≤50 years	16	13			15	14		
Sex	Female	23	10	0.5701	1.3 (0.50, 3.53)	20	13	0.6411	0.8 (0.31, 2.06)
	Male	26	15			27	14		
Organ failure	Present	16	3	0.0544	3.6 (0.93, 13.66)	15	4	0.1050	2.7 (0.79, 9.19)
	Absent	33	22			32	23		
Malignancy	Present	7	1	0.1777	4.0 (0.47, 34.49)	7	1	0.1356	4.6 (0.53, 39.17)
	Absent	42	24			40	26		
Time elapsed	> 24 h	43	22	0.9757	1.0 (0.22, 4.29)	43	22	0.2048	2.4 (0.60, 10.02)
	≤24 h	6	3			4	5		
Origin	Non colonic	37	22	0.2062	0.4 (0.11, 1.66)	35	24	0.1374	0.4 (0.09, 1.43)
	Colonic	12	3			12	3		
Extension	Generalized	33	17	0.9547	1.0 (0.35, 2.72)	34	16	0.2472	1.8 (0.66, 4.88)
	Localized	16	8			13	11		
Exudates	Fecal	6	0	0.1004	1.9 (0.70, 4.95)	5	1	0.0710	2.4 (0.92, 6.39)
	Purulent	25	12			26	11		
	Clear	18	13			16	15		
Survival	Survivor	19	3	**0.017**	**4.6 (1.22, 17.67)**	18	4	**0.0334**	**3.6 (1.06, 12.01)**
	Non-survivor	30	22			29	23		
BMI	Overweight/ obese	22	12	0.8001	0.9 (0.34, 2.32)	22	12	0.8443	1.1 (0.42, 2.85)
	Normal/ underweight	27	13			25	15		

### Cytokines

The presence of mutant allele A (21/34) with respect to -2548 polymorphism was associated with differences in serum *LEP *(*P *= 0.013), and IL-10 (*P *= 0.0001), compared to the wild type allele G. The presence of mutant allele G (22/34) with respect to 223 polymorphism showed a difference in serum *LEP *(*P *< 0001), C-RP (*P *= 0.033), and IL-10 (*P *= 0.043), compared to the wild type allele A. But, of the aforementioned *P*-values, only serum *LEP *in the presence of the 223 polymorphism fell in the 95% CI (Table 5).

**Table 5:** Relationship between polymorphisms and cytokine/acute phase reactant studied in patients with non-appendicular secondary peritonitis.

	-2548				223			
Cytokine/Acute phase reactant	Carriers *n *= 21	Non-carriers *n *= 13	*P*	(IC95)	Carriers *n *= 22	Non-carriers *n *= 12	*P*	(IC95)
Leptin	4.59 ng/ml	8.32 ng/ml	0.013	-3.52, 11.04	2.53 ng/ml	12.39 ng/ml	**< 0.0001**	3.176, 16.52
								
IL-6	8.35 pg/ml	9.10 pg/ml	0.684	-1.28, 2.76	8.82 pg/ml	8.27 pg/ml	0.130	-1.49, 2.61
								
TNF	21.80 pg/ml	21.75 pg/ml	0.483	-9.69, 9.79	23.06 pg/ml	19.43 pg/ml	0.598	-6.19, 13.45
								
CRP	160.39 μg/100 ml	150.52 μg/100 ml	0.756	-24.47, 44.21	165.58 μg/100 ml	140.18 μg/100 ml	0.033	-8.49, 59.29
								
IL-10	17.95 pg/ml	8.51 pg/ml	0.0001	-7.95, 26.83	13.87 pg/ml	15.21 pg/ml	0.043	-16.66, 19.34
								
IL-13	12.71 pg/ml	19.60 pg/ml	0.910	-4, 56, 18.34	17.55 pg/ml	11.30 pg/ml	0.337	-5.45, 17.95

If we compare the means of the study subjects with cytokine levels (*n *= 34) with those without these measurements (*n *= 40) there are no statistically significant differences in age (*P *= 0.197), sex (0.483), BMI classification (*P *= 0.607), the presence of -2548 polymorphism (*P *= 0.472) or 223 polymorphism (*P *= 1.000). These subsamples are equivalent, because all enrolled into the study in the manner stated in the Methods section.

## Discussion

The population base of the Hospital General de Durango, SSD is comprised mainly of underprivileged patients that lack access to social security medical institutions, which helps to explain the malnutrition observed. In a gross nutritional assessment, we expected no differences between cases and controls. Although there were significant differences between survivors and non-survivors in total proteins, albumin and the A/G ratio, there was no significant difference in BMI, making both groups comparable.

The differences in MPI severity between survivors and non-survivors was expected because the index is prognostic, and as the score increases, so does the probability of a poor outcome.

The lack of Hardy-Weinberg equilibrium in the *LEPR *223 polymorphism may be due to a low recombination rate, different to that present in *LEP *gene polymorphism -2548, within the promoter region.

Based on previous studies related to *LEP *and *LEPR *polymorphisms, differences in LEP levels, and physiological consequences were expected. Due to the fact that *LEP *gene polymorphism -2548 is located in the promoter region, it is associated with a low expression of LEP, and, consequently, low serum concentrations [9]. *LEPR *223 polymorphism, found in the exonic region, causes problems with ligand-receptor binding [12]. Both receptors have been related with obesity, non-Hodgkin's lymphoma, non-insulin-dependent diabetes mellitus, essential hypertension, and resistance to insulin [10,12].

Of the variables studied, the -2548 polymorphism superseded all other variables recognized as adverse factors for an unfavourable outcome. In the case of the 223 polymorphism, only coexistent malignancy surpassed it among the adverse factors studied (Table 4). It stands out that, other than outcome, none of the variables studied was found to be significantly associated to either polymorphism. As far as we know, this is the first report of the association between *LEP *and *LEPR *genetic polymorphisms and death, in any form of sepsis.

The *LEPR *gene 223A > G polymorphism is expected to have an impact on the binding of the hormone to its receptor. A change at the binding site may affect the union between the ligand and its receptor, modifying the intracellular signaling events, thereby influencing the expected physiologic response. The low concentration of LEP and a subsequent loss of its protective effects then has an impact on survival, but the presence of the polymorphism may contribute to this problem, explaining the low specificity of this biomarker, in accordance to a carrier or non-carrier state.

The fact that the poor outcome of patients with -2548 and 223 polymorphisms appears to be due to lower serum levels of LEP coincides with our previous report of a high risk of death in patients with surgically confirmed secondary peritonitis with low serum LEP levels. As suggested by these results, the threshold of 6.6 ng/ml (confirmed in this sample) should be taken as a predictive marker, as we stated in that publication [8]. LEP increases T-helper cells-1 (Th1) and suppresses Th2 cytokine production, hence its rise in sepsis promotes the host's pro-inflammatory response against its aggressors, and the lack of increase observed favours an anti-inflammatory pattern with an unfavourable outcome [25].

Bornstein reported an increase of LEP in sepsis, with lower levels among non-survivors [26,27]. A recent study found that serum LEP is a powerful biomarker that helps to differentiate sepsis from the non-infectious systemic inflammatory response syndrome in critically ill patients [28]. These findings, along with the aforementioned threshold, and our present results, underline the relevance of LEP in sepsis [8].

As to the possible mechanisms by which an insufficient increase in LEP may relate to mortality and systemic inflammation during sepsis, in a murine model, Tschöp *et al. *suggest that LEP deficiency leads to impaired bacterial clearance at the site of infection, and LEP replacement improves the immune response and reduces organ damage in sepsis. These authors suggest that the protective effect of LEP in sepsis occurs through CNS signalling that coordinates an appropriate immune response [29,30]. Shapiro *et al.*, in mouse endotoxemia and caecal ligation puncture models of sepsis, confirmed a rise in LEP levels in sepsis. Nonetheless, in their studies, exogenously administered LEP increased mortality, resulting in up-regulation of inflammatory mediators during endotoxemia and LEP-mediated endothelial dysfunction [31]. An explanation for these differences may be found in the manner in which each author administrated LEP. Tschöp *et al. *managed LEP replacement with intra-peritoneal injection, intra-cerebro-ventricular or peripheral mini-pumps (subcutaneous), while Shapiro *et al. *used osmotic pumps implanted into the abdominal cavity, which might not be the best choice if the model of sepsis is peritoneal.

The conflicting literature on LEP and sepsis is related to the type of patient studied, the source of the sepsis, and time of sampling, which is related to the complex dynamics of cytokines [32-34]. These factors must be taken into account so as not to confuse conflicting results with differences in methodology and the design, which is chosen in relation to the purpose of the study. A useful question to ask when reading papers related with research on LEP may be: does this paper study LEP role as a classic hormone in the regulation of body mass or its paper as a cytokine in the systemic inflammatory response?

The sum of these findings underlines the need to further study the role of LEP in sepsis.

Even though the results of the association between outcome and the polymorphism studied are significant, in spite of limitations in size of the sample, further studies with a larger group may show associations with clinical variables, cytokines or acute phase reactants not seen here.

## Conclusions

The -2548G > A *LEP *gene, and 223A > G *LEPR *gene polymorphisms are associated with death in patients with surgically confirmed secondary peritonitis of non-appendicular origin. This association is stronger than many known risk-factors related to peritonitis severity, and is independent of BMI. The patho-physiological mechanism is believed to be related to an insufficient increase in the elevation of serum LEP levels during sepsis.

## Key messages

*• LEP *Gene -2548G > A, and the *LEPR *Gene 223A > G polymorphisms increase the risk of death in secondary peritonitis with an odds ratio of 4.64 and 3.57, respectively.

• These polymorphisms are related with an insufficient increase of LEP serum levels in peritonitis.

• The threshold of 6.6 ng/ml in serum LEP levels is confirmed as an outcome marker.

## Abbreviations

BMI: Body Mass Index; C-RP: C-reactive protein; IL: interleukin; LEP: leptin; LEPR: leptin receptor; LPS: lipopolysaccharide; MOF: multiple organ failure; MPI: Mannheim Peritonitis Index; OR: Odds ratios; PCR: polymorphism chain reaction; RFLPs: restriction fragment length polymorphism; SSD: Secretaría de Salud de Durango; TNF-α: tumour necrosis factor alpha

## Competing interests

The authors declare that they have no competing interests.

## Authors' contributions

RB and JPS conceived and designed the study. JPS and GAL collected the data. VL and GAL carried out the molecular genetic studies and immunoassays. AT, VL and RB performed the statistical analysis and drafted the manuscript. RB and VL wrote the paper. All authors read and approved the final manuscript
